# Supplementary material for: Unlocking the potential of biogas systems for energy production and climate solutions in rural communities
Source: Nat Commun. 2024 Jul 13;15:5900. doi: 10.1038/s41467-024-50091-9 (PMC11246535; doi:10.1038/s41467-024-50091-9)
Supplement: Supplementary file 1 — Supplementary information [file 41467_2024_50091_MOESM1_ESM.pdf]

Supplementary information

Unlocking the potential of biogas systems for energy production and climate solutions in rural communities

Supplementary Tables

Supplementary Table 1 Basic parameters of the five observed community biogas production and distribution system (CBPD) in Chinese rural areas

Village name	Number of biogas supply customers	Average value of customers			Biogas consumption (m^3 d^{-1})			Biogas production (m^3 d^{-1})			CPR (%)	Monitoring period
		Family size (persons)	Home time (month yr^{-1})	Age (yr)	Max	Min	Mean	Max	Min	Mean		
Longdong	35	3.0	10.8	51.1	20.4	5.1	11.6	40.8	2.6	14.7	78.0	Oct. 18, 2017- Apr. 5, 2019
Taikang	47	4.0	9.3	46.7	37.6	8.1	18.0	43.1	9.0	21.6	65.2	Dec. 12, 2017- Apr. 29, 2019
Guangping	60	4.3	10.0	44.6	76.6	21.2	43.0	57.9	18.6	36.1	73.4	Aug. 8, 2017- Oct. 29, 2018
Changping A	65	3.3	9.7	46.8	43.6	15.4	27.2	66.7	12.2	33.3	75.8	Jun. 23, 2018- Apr. 29, 2019
Changping B	120	6.5	9.5	53.8	79.9	19.4	53.3	99.8	32.1	63.1	70.2	Mar. 1, 2018- Apr. 29, 2019

The five villages are located on the vicinity of Deyang City, Sichuan province, China. The energy sources used by rural inhabitants include firewood, biogas, petrol gas, electricity, and solar energy for civilian energy consumption. From a least-cost perspective, the direct use of biogas as fuel seemed more reasonable than petrol gas, as the stability of biogas supply met the customers' requirement of energy service, and the price of biogas use was 0.084 Chinese Yuan MJ^{-1} compared with approximately 0.112 Chinese Yuan MJ^{-1} for petrol gas.

Supplementary Table 2 Parameters of the proposed community biogas production and distribution system (CBPD) for state-of-the-art performance analysis

Parameter	Value
Fermented volume (m^3)	100
Fermentation temperature (℃)	27
Total solid content of feedstock content (%)	10
Average OLR (kg wet weighted material d^{-1})	1667
Thickness of insulation (m)	0.1
Absorption of solar radiation by reactor surface	0.79
Thermal conductivity of insulation ($W\ m^{-1}$ ℃$^{-1}$)	0.42
Surface area of roof (m^2)	23.9
Surface area of wall (m^2)	80.1
Surface area of ground (m^2)	20.4

Supplementary Table 3 Operational parameters of a combined heat and power generation unit (CHP) and its greenhouse gas (GHG) emission of one customer's biogas usage

Average biogas consumption rate ($m^3\ d^{-1}$ $customer^{-1}$)	Electricity efficiency [1] (%)	Lower heating value of biogas ($MJ\ m^{-3}$)	Emission factor for generated electricity connecting to grid [2] (kg $CO_2eq\ kWh^{-1}$)	GHG emission of CHP * (kg $CO_2eq\ d^{-1}$ $customer^{-1}$)
0.427	30 (20—42)	21.54	0.2135—0.4819	(-0.109)—(-0.517)

*The GHG emission mitigation values were lower than that of upgraded community biogas production and distribution system deployment in all Chinese provinces.

Supplementary Table 4 Comparisons of upgraded community biogas production and distribution system (CBPD) with existing biogas systems for implementation

Item	Main biogas usage pattern	Practical challenge	Methane emission	Scalability	Prospects
Household digester	A conventional fuel for cooking or illumination in general situations.	The operation is dependent on the extent of livestock breeding in each household, and declines in breeding and poor energy services are the direct reasons for its discontinuation[1].	It is often poorly managed and lacking a proper biogas distribution system. The available information in developing countries indicates that emissions may be as high as 40% [2].	Biogas production and consumption rates are out-of-step, and the scalable feasibility is limited.	As the popularization of the use of commercial energy, intermittent biogas supply and low consumption-to-production ratio (CPR) would result in the dis-adoption increasing.
Medium- and large-scale biogas plant	Power or biomethane generation	Its wide deployment would face various challenges on economic feasibility and high level operation requirements in developing areas.	Methane emission could reach 10% cumulative production during the biogas production and distribution chain[3,4].	Depending on feedstock logistics and distributing feasibility of electricity grid net and natural gas pipe net.	The large-scale livestock farms and agricultural modernization would be beneficial for its development, but carbon mitigation contribution is relatively low owing to the conversion and distribution chain.
Community size biogas plants	A conventional fuel for cooking, bathing, or illumination	Operational level is the main limiting factor of a stable energy supply, and efficient methane control is the main barrier for carbon mitigation.	Methane emission is reported as 23.6% [5].	Technical barrier is the main obstacle to acquire the co-benefits.	It can provide stable and clean biogas for rural inhabitants' biogas usage; another usage is limited.
Upgraded CBPD	Not only direct biogas supply-on-demand for cooking, heating, and so on; but also converted energy generation on schedule, such as electricity.	Inaccurate pre-estimation of combined usages, and hard to reach sophisticated operation training.	CPR is expected to be close to 1.	Scalability depends on biogas consumption of communities and accuracy of data-driven characterization	High expectation of broad application to achieve energy equality and carbon emissions reduction in rural communities.

Supplementary Table 5 Rates of methane production from manure and domestic gas demand in the rural community (RPD) in 31 provinces of the Chinese mainland

Item	Province	Methane demand, (billion m^3 CH_4 yr^{-1})	RPD
1	Beijing	0.10	1.33
2	Tianjin	0.07	2.35
3	Hebei	1.00	2.31
4	Shanxi	0.49	5.85
5	Inner Mongolia	0.31	4.51
6	Liaoning	0.44	6.80
7	Jilin	0.34	5.28
8	Heilongjiang	0.43	4.56
9	Shanghai	0.12	0.88
10	Jiangsu	0.82	2.47
11	Zhejiang	0.70	1.66
12	Anhui	0.91	3.76
13	Fujian	0.45	3.06
14	Jiangxi	0.57	4.42
15	Shandong	1.34	4.41
16	Henan	1.44	3.82
17	Hubei	0.74	4.64
18	Hunan	0.95	4.91
19	Guangdong	0.97	4.29
20	Guangxi	0.73	4.85
21	Hainan	0.11	4.79
22	Chongqing	0.40	3.36
23	Sichuan	1.33	4.23
24	Guizhou	0.59	3.04
25	Yunnan	0.69	4.67
26	Xizang	0.05	15.77
27	Shaanxi	0.51	1.93
28	Gansu	0.37	2.71
29	Qinghai	0.07	10.90
30	Ningxia	0.08	2.81
31	Xinjiang	0.32	2.95

Supplementary Table 6 Collective carbon emissions of rural inhabitants' energy use

Item	Structural countryside inhabitants' energy use	Percentage (%)	Emission factors (kg CO_2eq MJ^{-1})	Collective emission of countryside inhabitants' energy use (kg CO_2eq MJ^{-1})
1	Coal	14.6	0.0908	
2	Oil	10.0	0.0590	
3	LPG	5.9	0.0590	
4	Gas	0.8	0.0532	
5	Electricity	31.5	0.1614	0.0739
6	Biogas	2.9	0	
7	Straw	11.7	0	
8	Firewood	19.5	0	
9	Solar	3.1	0	

Supplementary notes

Supplementary Note 1. Effect of feeding time point on the requirements of storage capacity and residue biogas held in a storage facility at the substrate loading site

Appropriate biogas storage capacity with sufficient biogas presetting (residue biogas) is the necessary condition for a guaranteed biogas supply. The requirements for storage capacity and its minimum residue biogas at different feeding time points are shown in Supplementary Fig. 1. We suggest that, in practice, feeding time point should be selected at the evening to optimize biogas supply for rural inhabitants' cooking, which can reduce the requirement for residue biogas amount and biogas storage capacity. Practically, once some other biogas consumption information is available, daily biogas consumption could be forecast on site, and a more accurate operational scheme could be refined according to the optimal biogas storage capacity.

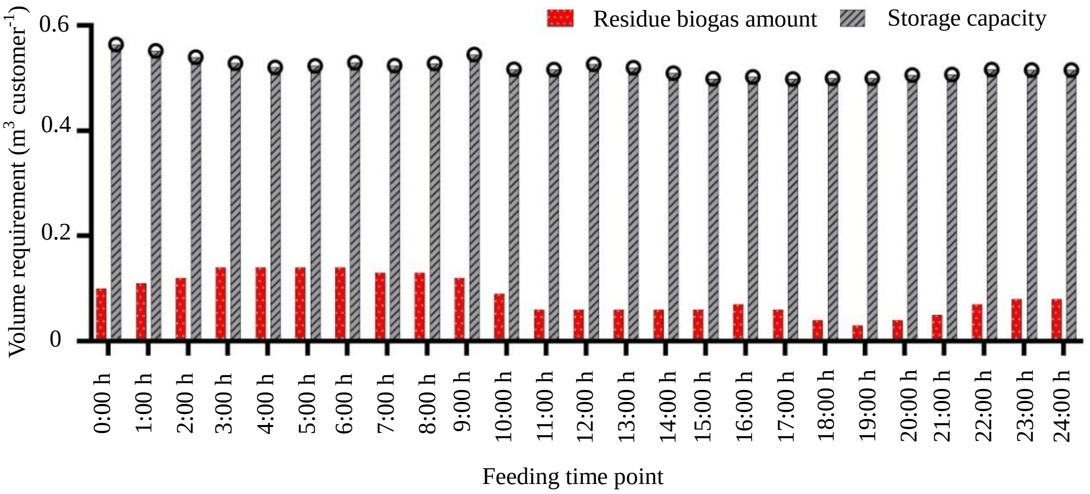

Supplementary Figure 1. Requirements of biogas storage volume and its minimum residue biogas at different feeding time points. The black circles represent the minimum volume requirement of biogas storage under each feeding time point to obtain consumption-to-production ratios of 1.

Supplementary Note 2 Outdoor solar-air temperature and thermal balance calculation of community biogas production and distribution system operation.

The expression of outdoor solar-air temperature (T_{st}) is calculated as Equation S1.

$$T_{st} = T_a + \frac{\alpha \cdot I}{h_{out}} - RF_t \mathrm{E}_{\mathrm{CBPD}} = 25 \cdot \left((\mathrm{X}(\triangle\ \mathrm{t})_{\mathrm{p}} - \mathrm{X}(\mathrm{t})_{\mathrm{p}}) - (\mathrm{X}(\triangle\ \mathrm{t})_{\mathrm{c}} - \mathrm{X}(\mathrm{t})_{c}) \right) \cdot \mathrm{r} \cdot \rho - (\mathrm{X}(\triangle\ \mathrm{t})_{\mathrm{c}} - (\mathrm{X}(\triangle\ \mathrm{t})_{\mathrm{c}} - \mathrm{X}(\mathrm{t})_{c}) \cdot \mathrm{r} \cdot \mathrm{ef} \quad \text{(S1)}$$

Where: T_{st} is the outdoor solar-air temperature, T_a is the outdoor air temperature, α is the absorption of solar radiation by the ground surface, I is the solar radiation intensity, h_{out} is the absorption of solar radiation by reactor surface, and RF_t is the long-wave radiative factor between the surface and environment.

The total heat required to maintain the fermentation temperature at a desired value is expressed as Equation S2.

$$Q_T = Q_i + Q_d + Q_g + Q_W \quad \text{(S2)}$$

Where: Q_T is total digester heat requirement at a time interval of $\triangle t$, Q_i is heat required to raise the feedstock to the fermentation temperature at a time interval of $\triangle t$, Q_d is heat loss through digester walls, bottom and top at a time interval of $\triangle t$, Q_g is heat loss due to the gas leaving digester at a time interval of $\triangle t$, Q_W is heat loss due to evaporation at a time interval of $\triangle t$. The detail calculation equation has been previously described [3].

Supplementary Note 3 The climate conditions of 10 cities and their detailed parameters

The small-scale biogas plants are likely to be most feasible in Chinese rural areas with fermentation volume of approximate 100 m^3, which could provide a possible solution to supply biogas for rural concentrated settlements, as the household number of countryside inhabitants is around 100 m^3 with a provision of 1 of biogas per customer per day [9]. In addition, the biogas production capacity could reach 100 m^3 or be at a low operational level, which can cover the most scenarios of usage with zero slipping for flexible production, not only for cooking, but also for heating, and so on. Thus, we selected a conventional community biogas production and distribution system of 100 m^3 fermented volume as the typical case to study the performance of carbon mitigation in this study. Certainly, a higher fermented volume could have a better performance due to a lower percentage of digester heating requirements for universal deployment. The 2015 data for annual ambient temperature and solar radiation intensity of 10 selected cities was downloaded from the Energyplus website. The calculation model was selected as cylindrical reactor of 5.1 m inside diameter [4], with uniform digester material and insulation material heat loss intensity properties. The digesters were heated with biogas combustion when the monthly outdoor solar-air temperature fell below 27℃ [5]. The parameters used in the daily net biogas supply calculation are shown in Supplementary Table 2.

Supplementary Note 4 The conditions assumed in scattered Chinese agricultural and rural communities for national deployment of upgraded community biogas production and distribution system (CBPD).

The assumed boundary conditions were the following.

ⅰ) Rural dwellers fully understand the benefits of health, energy conservation, and the environment as illustrated by the wide application of household biogas digesters in recent years [6].

ⅱ) The increased income of countryside inhabitants will encourage them to pursue the convenient fuel and achieve higher living standards [7], and the use of commercial biogas with high quality supply services is an ideal choose.

ⅲ) The nuclear family is the fundamental unit for livestock farming systems running [8]. It is possible for upgraded CBPD to quickly and efficiently collect feedstock due to concentrated livestock and poultry farms. The settlement pattern in rural areas has changed from a decentralized pattern of communities to a centralized one with “integrated construction of urban-rural areas”, which is beneficial to minimize the biogas distribution investment and cost [9].

ⅳ) Biogas is to be the most likely future choice of fuel in rural communities as cooking, heating, and commercial energy for agricultural enterprises [10].

Supplementary references

1. Wellinger, A., Murphy, J. & Baxter, D. The Biogas Handbook. (Woodhead Publishing Limited, Cambridge, 2013).
2. MEE. 2019 Baseline Emission Factors Regional Power Grids China. (MEE, 2020).
3. Li, Y., Yan, B., Qin, Y., Shi, W. & Yan, J. Analysis of the types of animal husbandry and planting that influence household biogas in rural China. *Journal of Cleaner Production* **332**, 130025 (2022).
4. Bluemling, B. & Visser I. D. Overcoming the "club dilemma" of village-scale bioenergy projects—The case of India. *Energy Policy* **63**, 18-25 (2013).
5. Bakkaloglu, S., Cooper, J. & Hawkes, A. Methane emissions along biomethane and biogas supply chains are underestimated. *One Earth* **5**, 724-736 (2022).
6. Wechselberger, V., Reinelt, T., Yngvesson, J., Scharfy, D., Scheutz, C., Huber-Humer, M. & Hrad, M. Methane losses from different biogas plant technologies. *Waste Management* **157**, 110-120 (2023).
7. Luo, T., et al. Reducing biogas emissions from village-scale plant with optimal floating-drum biogas storage tank and operation parameters. *Applied Energy* **208**, 312-318 (2017).
8. Luo, T., Meng, X., Long, E., Mei, Z. & Guo, X. Wall thermal load analysis and engineering operation of ground type biogas tank. *Acta energiae solaris sinica* **38**, 2069-2076 (2017).
9. Luo, T., et al. A case study assessment of the suitability of small-scale biogas plants to the dispersed agricultural structure of China. *Waste & Biomass Valorization* **7**, 1131–1139 (2016).
10. Liu, J., Zhou, X., Wu, J., Gao, W. & Qian, X. Heat transfer analysis of cylindrical anaerobic reactors with different sizes: a heat transfer model. *Environmental Science and Pollution Research* **24**, 23508-23517 (2017).
11. Liu, Y., Chen, Y., Li, T., Wang, D. & Wang, D. Investigation on the heat loss characteristic of underground household biogas digester using dynamic simulations and experiments. *Biosystems Engineering* **163**, 116-133 (2017).
12. Chen, Q., Yang, H., Liu, T. & Zhang, L. Household biomass energy choice and its policy implications on improving rural livelihoods in Sichuan, China. *Energy Policy* **93**, 291-302 (2016).
13. Wu, S., Zheng, X. & Wei, C. Measurement of inequality using household energy consumption data in rural China. *Nature Energy* **2**, 795-803 (2017).
14. Yang, F., et al. The agriculture and society in the Yiluo River Basin: Archaeobotanical evidence from the Suyang Site. *Frontiers in Earth Science* **10**, 885837 (2022).
15. Hales, D. Renewables 2018 Global Status Report. (Renewable Energy Policy, Network, 2018).
