# Supplementary material for: Unlocking the potential of biogas systems for energy production and climate solutions in rural communities
Source: Nat Commun. 2024 Jul 13;15:5900. doi: 10.1038/s41467-024-50091-9 (PMC11246535; doi:10.1038/s41467-024-50091-9)
Supplement: Supplementary file 3 — Reporting Summary [file 41467_2024_50091_MOESM3_ESM.pdf]

nature portfolio

Corresponding author(s): Bo Shen

Last updated by author(s): Mar 3, 2024

Reporting Summary

Nature Portfolio wishes to improve the reproducibility of the work that we publish. This form provides structure for consistency and transparency in reporting. For further information on Nature Portfolio policies, see our Editorial Policies and the Editorial Policy Checklist.

Statistics

For all statistical analyses, confirm that the following items are present in the figure legend, table legend, main text, or Methods section.

n/a	Confirmed	
☐	☒	The exact sample size (n) for each experimental group/condition, given as a discrete number and unit of measurement
☐	☒	A statement on whether measurements were taken from distinct samples or whether the same sample was measured repeatedly
☐	☒	The statistical test(s) used AND whether they are one- or two-sided<br>*Only common tests should be described solely by name; describe more complex techniques in the Methods section.*
☐	☒	A description of all covariates tested
☐	☒	A description of any assumptions or corrections, such as tests of normality and adjustment for multiple comparisons
☐	☒	A full description of the statistical parameters including central tendency (e.g. means) or other basic estimates (e.g. regression coefficient) AND variation (e.g. standard deviation) or associated estimates of uncertainty (e.g. confidence intervals)
☒	☐	For null hypothesis testing, the test statistic (e.g. F, t, r) with confidence intervals, effect sizes, degrees of freedom and P value noted<br>*Give P values as exact values whenever suitable.*
☒	☐	For Bayesian analysis, information on the choice of priors and Markov chain Monte Carlo settings
☒	☐	For hierarchical and complex designs, identification of the appropriate level for tests and full reporting of outcomes
☐	☒	Estimates of effect sizes (e.g. Cohen's d, Pearson's r), indicating how they were calculated

Our web collection on statistics for biologists contains articles on many of the points above.

Software and code

Policy information about availability of computer code

Data collection	Hourly biogas production, hourly biogas consumption, and their methane contents of the five selected CBPDs were measured using transit-time ultrasonic gas flow meters (TY1030, TianYu, Wuhan, China), which were easy to install with minimal or no disruptions to the flow, and had several vital advantages, such as high accuracy and a wide range ratio.
Data analysis	Origin 9.0 and Excel 2010 were used for all statistical assessments.

For manuscripts utilizing custom algorithms or software that are central to the research but not yet described in published literature, software must be made available to editors and reviewers. We strongly encourage code deposition in a community repository (e.g. GitHub). See the Nature Portfolio guidelines for submitting code & software for further information.

Data

Policy information about availability of data

All manuscripts must include a data availability statement. This statement should provide the following information, where applicable:

- Accession codes, unique identifiers, or web links for publicly available datasets
- A description of any restrictions on data availability
- For clinical datasets or third party data, please ensure that the statement adheres to our policy

All relevant data related to this article are documented in source data available at the figshare repository.

Research involving human participants, their data, or biological material

Policy information about studies with human participants or human data. See also policy information about sex, gender (identity/presentation), and sexual orientation and race, ethnicity and racism.

Reporting on sex and gender	All relevant data related to this article are documented in source data available at the figshare repository.
Reporting on race, ethnicity, or other socially relevant groupings	All relevant data related to this article are documented in source data available at the figshare repository.
Population characteristics	All relevant data related to this article are documented in source data available at the figshare repository.
Recruitment	All relevant data related to this article are documented in source data available at the figshare repository.
Ethics oversight	All relevant data related to this article are documented in source data available at the figshare repository.

Note that full information on the approval of the study protocol must also be provided in the manuscript.

Field-specific reporting

Please select the one below that is the best fit for your research. If you are not sure, read the appropriate sections before making your selection.

☐ Life sciences ☐ Behavioural & social sciences ☒ Ecological, evolutionary & environmental sciences

For a reference copy of the document with all sections, see nature.com/documents/nr-reporting-summary-flat.pdf

Ecological, evolutionary & environmental sciences study design

All studies must disclose on these points even when the disclosure is negative.

Study description	All relevant data related to this article are documented in source data available at the figshare repository.
Research sample	Exploring with rural residential biogas supply. The five villages are located on the vicinity of Deyang City, Sichuan province, China. The detail parameters are listed in Supplementary Table 1 Basic parameters of the five observed community biogas production and distribution system (CBPD) in Chinese rural areas.
Sampling strategy	All data, (X1, X2,..., Xn), were restructured to achieve schema integration of the feeding interval data, including steps such as splitting, merging, folding, and unfolding, to resolve and overlap conflicting representations. The measured biogas production set and biogas consumption set were represented by sets Sp and Sc, respectively; S = {X1, X2,..., Xn}. The daily biogas production set and biogas consumption set were represented by sets Xp and Xc, respectively; S = X(n, t) = {x1, x2,..., x24}. The data collected were used as the respective X at day n and hour t.<br>To detect and remove the sets of errors and inconsistencies, a detailed data analysis was performed. The cleaning process on the given dataset made the following assumptions.<br>1. If any value of Xp was less than 0.1 or more than five times the daily average value, the value was either considered to be an outlier or biogas production did not follow the normal distribution, and day Xp was removed from the dataset.<br>2. If any value of Xc between 1 am–4 am was more than 0.5 m3 h-1 customer-1, it meant that biogas leakage or an inaccurate measurement may have occurred. Furthermore, if the daily average value of any Xc was two times higher or 0.5 times lower than that of the previous or following day, the data for the daily biogas consumption rate was considered to be an outlier, and the day Xc was removed from the dataset.<br>3. Finally, only when day Xp and Xc were both in the dataset, could the values be considered to be quality data; otherwise, the single X value was deleted.
Data collection	Hourly biogas production, hourly biogas consumption, and methane content of the five selected community biogas production and distribution systems were measured using transit-time ultrasonic gas flow meters.
Timing and spatial scale	All data were collected for the time period Aug. 8, 2017- Apr. 29, 2019 from the five selected community biogas production and distribution systems.
Data exclusions	no data were excluded from the analyses.
Reproducibility	Biogas flow fitting was taken with five actual community biogas production and distribution systems in Chinese rural areas to investigate the status-quo of current operations. Sensitive analyses was carried out using the established data to validate the reproducibility.
Randomization	We modelled biogas flow with five actual community biogas production and distribution systems. The randomization was not relevant to our our study except that when the application was on-site. In that case, sensitive analyses using the established data.
Blinding	As the paper focus on the cases studies and the improved design, the sensitive analyses and coordinated scenarios are investigated. Therefore, blinding test was not relevant to your study as the special experiments is not included.

Did the study involve field work? [x] Yes [ ] No

Field work, collection and transport

Field conditions	The five villages are located on the vicinity of Deyang City, Sichuan province, China. The energy sources used by rural inhabitants include firewood, biogas, petrol gas, electricity, and solar energy for civilian energy consumption. From a least-cost perspective, the direct use of biogas as fuel seemed more reasonable than petrol gas, as the stability of biogas supply met the customers' requirement of energy service, and the price of biogas use was 0.084 Chinese Yuan MJ-1 compared with approximately 0.112 Chinese Yuan MJ-1 for petrol gas.. More detailed parameters could be found in Supplementary Table 1.
Location	The five villages are located on the vicinity of Deyang City, Sichuan province, China.
Access & import/export	This study does not involve related content.
Disturbance	Each CBPD was equipped with two meters with dehydration systems, which were used to avoid dew formation. All meters were calibrated and validated at test facilities for biogas measurement every 6 months.

Reporting for specific materials, systems and methods

We require information from authors about some types of materials, experimental systems and methods used in many studies. Here, indicate whether each material, system or method listed is relevant to your study. If you are not sure if a list item applies to your research, read the appropriate section before selecting a response.

Materials & experimental systems

n/a	Involved in the study	
[x]	[ ]	Antibodies
[x]	[ ]	Eukaryotic cell lines
[x]	[ ]	Palaeontology and archaeology
[x]	[ ]	Animals and other organisms
[x]	[ ]	Clinical data
[x]	[ ]	Dual use research of concern
[x]	[ ]	Plants

Methods

n/a	Involved in the study	
[x]	[ ]	ChIP-seq
[x]	[ ]	Flow cytometry
[x]	[ ]	MRI-based neuroimaging

Plants

Seed stocks	This study does not involve related content.
Novel plant genotypes	This study does not involve related content.
Authentication	This study does not involve related content.
